# Current Understanding of the Right Ventricle Structure and Function in Pulmonary Arterial Hypertension

**DOI:** 10.3389/fphys.2021.641310

**Published:** 2021-05-28

**Authors:** Danial Sharifi Kia, Kang Kim, Marc A. Simon

**Affiliations:** ^1^Department of Bioengineering, University of Pittsburgh, Pittsburgh, PA, United States; ^2^Division of Cardiology, Department of Medicine, University of Pittsburgh, Pittsburgh, PA, United States; ^3^Heart and Vascular Institute, University of Pittsburgh Medical Center, Pittsburgh, PA, United States; ^4^Pittsburgh Heart, Lung, Blood and Vascular Medicine Institute, University of Pittsburgh - University of Pittsburgh Medical Center, Pittsburgh, PA, United States; ^5^McGowan Institute for Regenerative Medicine, University of Pittsburgh, Pittsburgh, PA, United States; ^6^Department of Mechanical Engineering and Materials Science, University of Pittsburgh, Pittsburgh, PA, United States; ^7^Center for Ultrasound Molecular Imaging and Therapeutics, University of Pittsburgh, Pittsburgh, PA, United States; ^8^Division of Cardiology, Department of Medicine, University of California, San Francisco, San Francisco, CA, United States

**Keywords:** right ventricle, biomechanics, pulmonary hypertension, remodeling, RV failure, structure, function, hemodynamics

## Abstract

Pulmonary arterial hypertension (PAH) is a disease resulting in increased right ventricular (RV) afterload and RV remodeling. PAH results in altered RV structure and function at different scales from organ-level hemodynamics to tissue-level biomechanical properties, fiber-level architecture, and cardiomyocyte-level contractility. Biomechanical analysis of RV pathophysiology has drawn significant attention over the past years and recent work has found a close link between RV biomechanics and physiological function. Building upon previously developed techniques, biomechanical studies have employed multi-scale analysis frameworks to investigate the underlying mechanisms of RV remodeling in PAH and effects of potential therapeutic interventions on these mechanisms. In this review, we discuss the current understanding of RV structure and function in PAH, highlighting the findings from recent studies on the biomechanics of RV remodeling at organ, tissue, fiber, and cellular levels. Recent progress in understanding the underlying mechanisms of RV remodeling in PAH, and effects of potential therapeutics, will be highlighted from a biomechanical perspective. The clinical relevance of RV biomechanics in PAH will be discussed, followed by addressing the current knowledge gaps and providing suggested directions for future research.

## Introduction

Clinically, pulmonary hypertension (PH) is defined as a condition with resting mean pulmonary artery pressures (mPAP) >20 mmHg (Simonneau et al., 2019). PH results in right ventricular (RV) pressure overload and leads to multi-scale adaptations in RV structure/function in response to the increased afterload. Although PH initiates from pulmonary vascular remodeling, progression to RV dysfunction and failure remains the main cause of mortality in PH patients (Voelke et al., 2011). The world health organization (WHO) classifies PH into 5 different groups (Simonneau et al., 2019): Group I: PH due to pulmonary arterial hypertension (PAH) or pre-capillary PH, Group II: PH due to left heart disease (PH-LHD), Group III: PH due to underlying lung disease/hypoxia, Group IV: Chronic thromboembolic PH, Group V: PH with multifactorial underlying cause or unclear mechanism. While PH-LHD remains the most common type of PH (Guazzi and Borlaug, 2012), limited data exists on RV biomechanics in the setting of PH-LHD (Simon et al., 2016; Zou et al., 2018; Philip et al., 2019; Bashline et al., 2020), while several biomechanical studies have evaluated RV function in PAH. Hence, the current review focuses on biomechanics of the RV in PAH – the precapillary phenotype of PH (Group I).

Pulmonary arterial hypertension has been shown to result in RV remodeling at different scales, from changes in organ-level hemodynamics to tissue stiffening, fiber reorientation, and altered myocyte contractility and mitochondrial energetics (Hill et al., 2014; Avazmohammadi et al., 2017a; Pewowaruk et al., 2018; Sharifi Kia et al., 2020). Mechanical stimuli play a key role in RV remodeling in response to pressure overload and biomechanical analysis of RV pathophysiology has drawn significant attention over the past years. Multi-scale biomechanical analysis frameworks have been employed to investigate the underlying mechanisms of RV remodeling in PAH (Wang et al., 2018; Avazmohammadi et al., 2019a) and have linked RV biomechanics to physiological function (Jang et al., 2017). Functional measures of RV systolic and diastolic mechanics have been found to be strong predictors of outcomes in PH (Trip et al., 2015; Vanderpool et al., 2015), indicating a potential for translation of biomechanical measures to clinical relevance (Jang et al., 2017). Recent work has provided important insights into the time-course of multi-scale remodeling events in PAH and has analyzed the potential of different therapeutic interventions to attenuate RV remodeling.

In this review, we discuss the recent progress in understanding the multi-scale biomechanics of the RV in PAH across different scales from organ-level function to tissue, fiber, and cellular-level mechanics. This will be followed by highlighting the potential of computational/*in silico* studies for multi-scale evaluation of adaptive vs. maladaptive PAH-induced remodeling events. Common techniques for biomechanical characterization of the RV at different scales will be summarized, followed by a discussion on recent progress in understanding the biomechanical effects of potential therapeutic interventions.

## Multi-Scale Biomechanics of RV Remodeling in PAH

### Animal Models of PAH

Clinical work has been focused on studying RV mechanics in human patients using invasive hemodynamics, cardiac magnetic resonance imaging (cMRI) and echocardiography. On the other hand, pre-clinical studies have employed animal models of PAH for mechanistic investigation of different RV remodeling events and the role of mechanical stimuli in these processes. The most commonly used animal models of PAH include: (1) PAH induced via vascular endothelial growth factor (VEGF) receptor blocker Sugen-5416 followed by exposure to hypoxia (SuHx), (2) Monocrotaline (MCT)-induced PAH, and (3) PAH induced via pulmonary artery banding (PAB) (Akazawa et al., 2020). The SuHx model results in similar angio-obliterative lesions to those developed in PAH patients, making this an ideal model to study pulmonary vascular mechanobiology in PAH. The MCT model has been widely used due to reproducibility and simplicity; however, development of myocarditis (Akhavein et al., 2007) remains a major limitation of this model. The PAB model is effective for studying the effects of PAH on RV remodeling, in the absence of confounding effects from pulmonary vascular remodeling or hypoxia (Bogaard et al., 2009; Borgdorff et al., 2013; Andersen et al., 2014; Hill et al., 2014). This makes the PAB model ideal to study RV remodeling due to PAH and potential benefits of therapeutic interventions, separate from alterations in pulmonary vascular resistance (PVR) or VEGF inhibition. A mild constriction via PAB results in adaptive RV remodeling with minor indications of RV fibrosis (Bogaard et al., 2009). However, recent studies have indicated that a sufficiently severe constriction induced by PAB can result in similar levels of elevation in RV pressures, dilation, hypertrophy, and fibrosis, to SuHx or MCT models, and may progress to maladaptive RV failure (Andersen et al., 2014; Akazawa et al., 2020). Also, worth noting that when referring to adaptive vs. maladaptive remodeling in this review, we define adaptive remodeling as changes associated with maintenance of physiological function (such as adequate oxygen delivery to tissues, maintained cardiac output and ejection fraction, etc.), while maladaptive remodeling refers to changes leading to reduction in contractile function, as defined by Frump et al. (2018). However, further research is needed to analyze the biomechanical effects of adaptive vs. maladaptive remodeling in relation to function, and characterization of the transition points from adaptation to maladaptation.

Different species have been used as PAH models, including mouse, rat, chicken, dog, sheep, pig, cow, monkey. Rat and mouse models of PAH have been the most commonly studied. Hypoxia induces similar pulmonary vascular changes across species, with increasing pulmonary vascular smooth muscle cell hypertrophy and perivascular inflammation in larger animals (Stenmark et al., 2009). Different strains of the same species can have different response to PAH models as well. Similarly, response to monocrotaline varies both within and across species, which is thought to be due to variable cytochrome P-450 related metabolism of monocrotaline (Stenmark et al., 2009). Mice have been particularly useful in studying genetics. Large animals such as cows and sheep have been used particularly for the study of high-altitude related PAH. Monkeys have been used for the study of human immunodeficiency virus (HIV) related PAH utilizing simian immunodeficiency virus. Lamb and piglets have been used for the study of PH related to congenital abnormalities (Colvin and Yeager, 2014).

### Effects of PAH on Organ-Level RV Mechanics and Function

**FIGURE 1:**
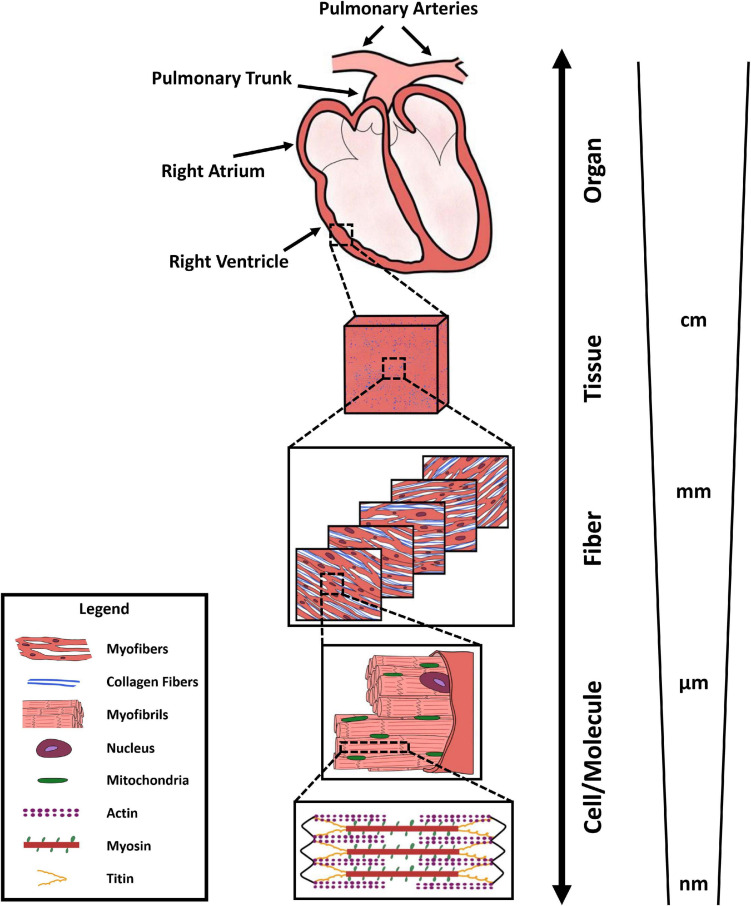
Multi-scale structure of the right ventricle, demonstrating organ-level anatomy, tissue-level organization (red/pink: myofiber content, blue: collagen content), transmural fiber-level architecture of collagen and myofibers, myofibril organization, mitochondrial content, and sarcomere-level actin-myosin interactions.

Due to the multi-scale structure of the RV, with specific features and function at different levels (Figure 1), different measurement techniques and metrics are used to evaluate RV biomechanics across different scales. At the organ level, RV function is often characterized by synchronous measurement of ventricular pressure and volume waveforms over a cardiac cycle, resulting in RV pressure-volume (P-V) loops. Additionally, multiple imaging modalities, including 2D and 3D echocardiography and cMRI, are employed for measurement of RV structure. P-V loop analysis helps evaluating RV peak, end-systolic, and end-diastolic pressures, in addition to end-systolic and end-diastolic volumes, stroke volume, cardiac output, and ejection fraction. Moreover, analyzing the rate of change in RV pressure waveforms enables assessment of load-dependent measures of RV contractility and relaxation, respectively measured by the maximum and minimum time derivatives of pressure ($\frac{d\,p}{d\,t}$_max_ and $\frac{d\,p}{d\,t}$_min_).

#### Organ-Level Remodeling in PAH

**FIGURE 2:**
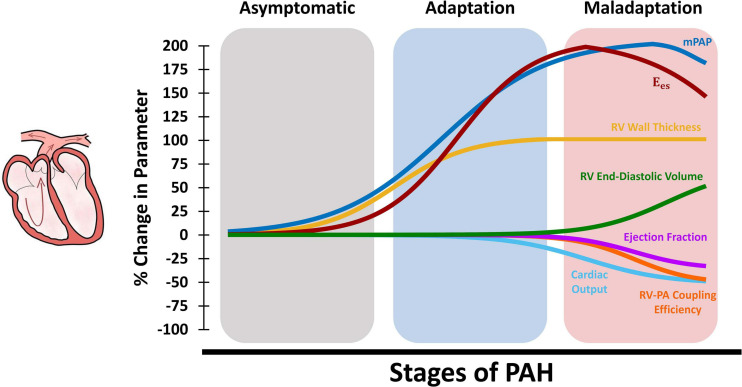
Schematic demonstration of changes in organ-level RV function at different stages of PAH. The RV initially responds to increased mean PA pressures (mPAP) via hypertrophy and increased RV wall thickness, which results in increased organ-level contractility (E_es_) and maintained cardiac output, ejection fraction, and RV-PA coupling (adaptation). Later in the process, RV hypertrophy reaches a plateau while mPAP is further elevated. The RV then undergoes dilation to maintain cardiac output which, together with other multi-scale remodeling events at the tissue, fiber and myocyte-level, leads to depressed organ-level contractility (E_es_), cardiac output, and ejection fraction. Sustained elevation in mPAP and decreased E_es_ results in reduced RV-PA coupling efficiency and RV-PA uncoupling (maladaptation). RV: Right ventricle; PAH: Pulmonary arterial hypertension; mPAP: Mean pulmonary artery pressures; PA: Pulmonary artery.

Right ventricular function is the most critical determinant of survival in PAH (Bogaard et al., 2009). Recent work indicates that RV dysfunction in PAH initiates at the organ-level, before progressing to impaired tissue and myocyte-level function (Wang et al., 2018). The RV undergoes multiple adaptive and maladaptive organ level remodeling events in response to PAH. As shown in the schematic in Figure 2, pulmonary vascular remodeling in PAH results in progressive elevation of mPAP. This, in turn, results in increased total PVR, as well as increased RV systolic and diastolic pressures. The RV initially responds to increased pressures in PAH by undergoing concentric hypertrophy (Figure 2; increased wall thickness), which helps reducing RV wall stress and results in increased organ-level contractility. Increased wall thickness results in maintained cardiac output and ejection fraction during the early stages of RV remodeling (Figure 2). However, with further progression of PAH, RV hypertrophy reaches a plateau (Wang et al., 2018) while PA pressures continue to rise (Vonk Noordegraaf et al., 2017). The RV then undergoes eccentric hypertrophy (RV dilation) in an attempt to maintain the stroke volume and cardiac output. However, RV dilation results in increased wall stress and RV oxygen consumption (Vonk Noordegraaf et al., 2017) and, together with other multi-scale remodeling events at the tissue and myocyte level, eventually leads to depressed organ-level contractility, reduced cardiac output and ejection fraction, and eventually RV dysfunction. It is also worth noting that while the upper/lower bounds of changes in the organ-level functional parameters presented in Figure 2 are based on previous extensive reports in the literature (Supplementary Table 1), the time-course of changes during the progression of PAH are illustrated based on a limited number of available longitudinal studies (Vonk Noordegraaf et al., 2017; Vélez-Rendón et al., 2018; Wang et al., 2018; Avazmohammadi et al., 2019a) and the authors’ speculation. Further long-term longitudinal biomechanical studies looking at the time-course of progression of PAH are warranted and would be highly beneficial to establish a better understanding of the effects of remodeling on functional mechanics of the RV. In a longitudinal study using a SuHx model of PAH, Wang et al. demonstrated preserved ejection fraction and increased organ-level contractility following 14 days of hypoxia (Wang et al., 2013, 2018). RV hypertrophy remained relatively constant after 14 days, while reduced ejection fraction and RV-PA coupling efficiency were observed at later time points (56 days of hypoxia), leading to RV dysfunction (Wang et al., 2018). However, myofilament contractile forces were elevated similarly at all different stages of PAH (with preserved Frank-Starling mechanism) suggesting the presence of RV dysfunction at the organ-level prior to myocyte-level dysfunction.

#### Effects of PAH on RV-PA Coupling

**FIGURE 3:**
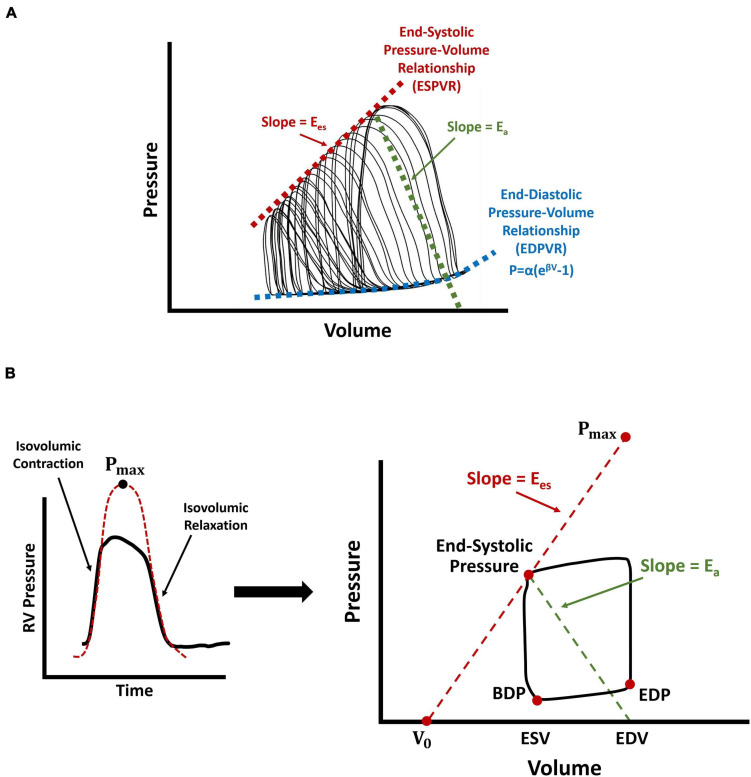
Schematic demonstration of different measurement techniques for characterization of RV end-systolic elastance, end-diastolic pressure-volume relationship (EDPVR), and RV-PA coupling efficiency. **(A)** RV preload reduction via vena caval occlusions in animal models, resulting in multiple P-V loops. **(B)** Single-beat estimation of RV end-systolic elastance using the “pressure method”, where P_max_ is characterized by the amplitude of a sinusoidal fit to the isovolumic contraction and relaxation portions of RV pressure waveforms. Here, V_0_ represents the volume axis intercept of the line between P_max_ and the end-systolic point of the P-V loop. RV-PA coupling efficiency is calculated using the ratio of $\frac{{\text{E}}_{\text{es}}}{{\text{E}}_{\text{a}}}$. Arrows show RV end-systolic elastance (red; E_es_), PA elastance (green; E_a_), and the nonlinear fit to the EDPVR (blue). For the equation used to fit the EDPVR, β represents the diastolic stiffness, α is a scaling factor, and P and V are respectively, end-diastolic pressure and volume data. RV: Right ventricle; PA: Pulmonary artery; P-V loop: Pressure-volume loop; BDP: Beginning-diastolic point; EDP: End-diastolic point; ESV: End-systolic volume; EDV: End-diastolic volume; EDPVR: End-diastolic pressure-volume relationship.

The dynamic supply demand mechanics of the RV and the pulmonary artery (PA) is another determinant of organ-level RV function (RV-PA coupling). A commonly used metric for evaluation of RV-PA coupling efficiency is the ratio of RV end-systolic elastance (E_es_) to PA elastance (E_a_), which conceptually is RV contractility per hydraulic load. In animal models, E_es_ (the load-independent measure of organ-level RV contractility) is assessed by preload reduction of the RV, via vena caval occlusion, which exposes the RV to a wide range of operational pressures and forms multiple P-V loops (Figure 3A). E_es_ is then calculated as the slope of a linear fit to the end-systolic pressure-volume relationship (ESPVR) (Sagawa et al., 1977; Sunagawa et al., 1983; Tabima et al., 2017). While recent work (Tello et al., 2020) has characterized E_es_ in human patients using multi-beat P-V loops, this remains a challenge in most centers, due to difficulties with simultaneous pressure and volume measurement and RV preload reduction in human subjects. Nevertheless, it is possible to estimate E_es_ using single-beat P-V data from right heart catherization in the clinical setting (Brimioulle et al., 2003). The single-beat pressure method estimates E_es_ using an additional parameter, P_max_, characterized by the peak amplitude of a sinusoidal fit to the isovolumic contraction and relaxation portions of RV pressure waveforms (Brimioulle et al., 2003; Figure 3B). E_es_ is then calculated using the ratio of (P_max_-end-systolic pressure) over stroke volume (Figure 3B). E_a_, on the other hand, is characterized similarly in the clinic and in animal models (Figure 3), using the ratio of RV end-systolic pressure to stroke volume (Sunagawa et al., 1983; Brimioulle et al., 2003; Vanderpool et al., 2015). The ratio E_es_/ E_a_ (RV-PA coupling) may be calculated by calculating the individual components, E_es_ and E_a_. However, alternatively, E_es_/ E_a_ may be estimated using the ratio of stroke volume/end-systolic volume which may be obtained from noninvasive imaging such as magnetic resonance imaging (Brewis et al., 2016) or 3D echocardiography (Jone et al., 2019). Other noninvasive echocardiography-derived estimates of RV-PA coupling, such as the ratio of tricuspid annular plane systolic excursion to estimated PA systolic pressures, have also been proposed which may provide for broader clinical translation of coupling concepts (Tello et al., 2019b). Many of these measures have been correlated with clinical outcomes in patients. For a detailed review of RV-PA coupling mechanics, please refer to the work by Tabima et al. (2017).

Right ventricular-PA coupling efficiency is a major determinant of outcomes in PH (Richter et al., 2020). Along with reduced cardiac output and ejection fractions, maladaptive RV remodeling in PAH, RV dilation, and depressed organ-level contractility leads to RV-PA uncoupling (Figure 2). End-stage PAH has been shown to result in decreased RV-PA coupling efficiency by ≈40-60% (Wang et al., 2018; Tello et al., 2019a, 2020; Sharifi Kia et al., 2020). Tello et al. employed cardiac magnetic resonance imaging (cMRI) and right heart catherization to study the RV-PA coupling reserve in PAH patients (Tello et al., 2019a). Reduction in RV-PA coupling (measured via single-beat analysis) was associated with increased end-diastolic volume index, PA stiffening, and reduced ejection fraction. Normal RV-PA coupling efficiency ranges from 1.5 to 2, while PAH patients with early signs of maladaptive remodeling demonstrated coupling efficiencies ranging from 0.89 to 1.09 (Tello et al., 2019a). Patients with severe progressive RV maladaptation showed coupling efficiencies around 0.56-0.61. A cutoff of 0.805 was defined for the onset of RV dilation and failure (defined as ejection fraction <35%), demonstrating a significant reserve for RV-PA coupling before progression to RV failure (Tello et al., 2019a). Another clinical study demonstrated that RV-PA coupling is an independent predictor of survival in PH (Vanderpool et al., 2015), which has been confirmed by several other studies (Brewis et al., 2016; Breeman et al., 2019; Jone et al., 2019; Hsu et al., 2020).

#### Effects of PAH on RV Diastolic Function

In addition to RV contractility and systolic function, PAH also affects RV diastolic function. Organ-level RV diastolic stiffness is characterized by the end-diastolic elastance (E_ed_) and the end-diastolic pressure-volume relationship (EDPVR), also obtained by measurement of RV P-V patterns at different loads via preload reduction (Figure 3A). Due to the nonlinear nature of passive RV mechanical properties, an exponential function is then fitted to the diastolic portion of the generated P-V loops, to characterize organ-level diastolic stiffness (β; Figure 3; Nikolic et al., 1988). Single-beat analysis methods have been proposed for regression of the exponential equation in Figure 3, using only three points: (1) the origin or V_0_ (Figure 3B), (2) the beginning diastolic point, and (3) the end-diastolic point. However, quality of fit and uniqueness of optimized parameters (due to a potential local minima) remains a major concern when characterizing an exponential function with limited number of points. Alternatively, other metrics such as the instantaneous end-diastolic elastance (E_ed_) may be used for biomechanical analysis of RV diastolic function (Gaynor et al., 2005; Jang et al., 2017), by evaluating the slope of the line tangent to the end-diastolic point of RV P-V loops.

Single-beat analysis of EDPVR in PAH patients revealed a significant increase in diastolic stiffness (β), which was correlated with metrics of disease severity such as RV stroke volume, 6-min walking distance and RA pressures (Rain et al., 2013). A significant increase in RV end-diastolic elastance (E_ed_) was observed in response to increased pressures via PAB, in a rat model of PAH (Jang et al., 2017). Moreover, E_ed_ was found to be positively correlated with β (Jang et al., 2017). Both E_ed_ and β were found to be significantly higher in patients with severe vs. mild PAH (Vanderpool et al., 2020). In a large animal PAB model, Gaynor et al. observed a significant increase in RV pressures and the load-dependent measure of RV contractility ($\frac{d\,p}{d\,t}$_max_), which was accompanied by increased RV elastance (Gaynor et al., 2005). While no differences in cardiac output were observed in response to PAB (indicating adaptive RV remodeling, potentially due to severity of the PAB procedure), there was a ≈2-fold increase in diastolic stiffness, which was attributed to impaired diastolic function (Gaynor et al., 2005). Taken together, these studies suggest that RV diastolic dysfunction may precede overt RV systolic decompensation and RV-PA uncoupling. It is worth noting that organ-level diastolic elastance derived from RV hemodynamic measures is load-dependent and does not compare to tissue-level stiffness measurements based on stress-strain mechanical testing data. The EDPVR has a nonlinear form and, therefore, diastolic elastance may increase in response to increased filling pressures, independent of structural RV remodeling (Levine, 1972). A structurally normal RV may demonstrate increased organ-level diastolic elastance in response to sufficiently elevated filling pressures (Levine, 1972).

#### Effects of PAH on RV Wall Stress and Contractile Strains

As the gold standard of ventricular afterload (Norton, 2001), organ-level left ventricular (LV) wall stress is often calculated by approximating the ventricular geometry by a sphere (or cylinder, depending on the remodeling stage), followed by stress calculations using the law of Laplace. However, the complex geometry of the RV complicates simple theoretical approximations of wall stress. While some studies have used the classic Laplace formulation for a sphere to approximate RV wall stress (Jang et al., 2017), others have developed an extension of the law of Laplace, considering the ellipsoidal geometry of the RV (Avazmohammadi et al., 2017a). Also, since the original Laplace formulation was proposed for thin-walled structures and RV hypertrophy and increased wall thickness in PAH may violate those assumptions, recent studies have employed a modified formulation for calculation of RV wall stress (Attard et al., 2019). Under normal hemodynamics, RV wall stress was found to be higher in the circumferential direction compared to the longitudinal (apex to base/outflow-tract) direction (Avazmohammadi et al., 2017a). PAH results in overall increased organ-level RV wall stress with a more significant elevation in the longitudinal direction [40 and 150% increase in circumferential and longitudinal directions, respectively (Avazmohammadi et al., 2017a)]. Wall stress analysis using combined pressure catheterization and cMRI in PAH patients (Richter et al., 2020) demonstrated a positive correlation between RV end-systolic wall stress and both diastolic RV elastance (E_ed_) and PA elastance (E_a_). In another clinical study, Attard et al. demonstrated that RV wall stress could be an independent predictor of all-cause mortality in a population of patients with PH of different etiologies (32% with PAH) (Attard et al., 2019).

Pulmonary arterial hypertension also affects the contractile mechanics of the RV free wall (RVFW). Alterations in RVFW contractile strains manifest early in the progression of PAH (Voeller et al., 2011). In animal models, *in vivo* contractile deformations can be characterized by placement of radiopaque markers on the RVFW, combined with biplane fluoroscopy (Chuong et al., 1991). Marker displacements can then be post-processed to measure RVFW fractional area reduction (measure of contractility). In an *in vivo* study on canine myocardium, Chuong et al. demonstrated that the outflow region of the RVFW undergoes higher fractional area reductions compared to the inflow and mid-ventricular regions (Chuong et al., 1991). Acute PA occlusion resulted in significant increases in RV peak pressures and organ-level contractility (measured by $\frac{d\,p}{d\,t}$_max_) by 106% and 48%, respectively. RVFW contractile deformations, however, demonstrated heterogenous regional variations. While RVFW contractility at the inflow and midventricular regions did not show any significant changes in response to acute pressure rise, the outflow region demonstrated decreased fractional area reduction by 22% (Chuong et al., 1991). The observed effects were attributed to differences in the embryological origin of the outflow region, compared to the inflow and mid-ventricular regions (March et al., 1962; Chuong et al., 1991). Echocardiography is another technique for *in vivo* evaluation of RVFW deformations, routinely used for clinical diagnosis of RV function. 2D ultrasound-based ventricular torsion and RVFW strain measurements have been employed for clinical assessment of regional and global RV function (Bossone et al., 2013). PAH was shown to result in reduced longitudinal RVFW peak systolic strains and strain rate, exhibiting associations with 1, 2, 3, and 4-year mortality in PAH patients (Sachdev et al., 2011). PAH patients with RVFW longitudinal peak systolic strains <19% demonstrate lower survival rates than those with strains >19% (Haeck et al., 2012). Reduced longitudinal systolic strains were also noted in experimental PAB, SuHx, and MCT models of PAH (Akazawa et al., 2020). Despite cost-effectiveness and wide availability of echocardiography, RV anatomy and positioning within the chest leads to reproducibility issues with ultrasound-based strain assessments (Bossone et al., 2013). Additionally, unlike the LV, circumferential and radial ultrasound-based strains are challenging to measure for the RVFW (Tadic et al., 2018). cMRI is a more accurate technique for *in vivo* RVFW strain measurements. In a large animal PAB model of PAH, cMRI demonstrated that severe pressure overload (250% increase in RV pressures) results in decreased RVFW contractility and minimum principal strains (measure of segmental shortening) at the basal level, within 10 weeks (Voeller et al., 2011). Moderate pressure overload (34% increase in RV pressures), however, did not show any significant effects on RVFW strains (Voeller et al., 2011).

#### Sex-Related Differences in RV Response to PAH

While PAH is more prevalent among females than males (Badesch et al., 2010), sex differences exist in the survival rates of PAH patients, with females demonstrating better outcomes (Jacobs et al., 2014; Lahm et al., 2014; Shen et al., 2020). The protective effects of the female sex hormone 17β-estradiol (estrogen; also known as E_2_) has been investigated in animal models of PAH (Liu et al., 2014, 2017b; Lahm et al., 2016). In response to similar levels of pressure overload induced via SuHx, female rats demonstrated better cardiac index, stroke volume and RV compliance. These effects were not present in ovariectomized female rats, while exogenous estrogen repletion lead to improved cardiac index and ejection fraction (Liu et al., 2014), and prevented RV-PA uncoupling (Liu et al., 2017b). Moreover, estrogen has been shown to prevent reduced PA compliance in PAH (Liu et al., 2014) and result in decreased PA systolic pressures (Liu et al., 2017a). Multi-scale studies suggest a direct effect from estrogen on RV remodeling via increasing RV contractility, in addition to indirect benefits from reduced PVR, reduced PA systolic pressures, and prevention of PA stiffening, leading to decreased RV afterload (Liu et al., 2014). Prospective analysis of RV function in PAH patients demonstrated better systolic adaptation (measured via E_es_) and RV-PA coupling efficiency in female patients (Tello et al., 2020), potentially contributing to the sex-related differences in survival rates of PAH patients. Despite similar levels of RV afterload (approximated by E_a_), median RV-PA coupling efficiency was higher than the 0.805 threshold (Tello et al., 2019a) in female patients, but not in males (Tello et al., 2020). An early clinical trial is now investigating the safety and efficacy of anastrozole, an aromatase inhibitor that prevents conversion of androgens to estrogen and 17β-estradiol (ClinicalTrials.gov Identifier: NCT03229499) based on provocative pilot data (Kawut et al., 2017).

#### Effects of PAH on Left Ventricular Organ-Level Mechanics

Pulmonary arterial hypertension primarily affects RV structure and function; however, RV and LV function are interdependent on one other and LV contraction assists with 20-40% of the systolic RV pressure rise (Santamore and Dell’Italia, 1998). Alterations in RV structure/function such as RV dilation and impaired contractility directly affect the organ-level biomechanics of the LV by reducing LV torsion and resulting in delayed peak torsion (Kaiser et al., 2020; Kheyfets et al., 2020). Isolated RV pressure overload in a PAB model resulted in reduced LV ejection fraction and stroke work, as well as LV atrophy (reduced wall thickness) (Kheyfets et al., 2020). Since the total work performed by LV contraction is shared between displacing blood within the systemic circulation and supporting RV contractions, reduced LV stroke work in this PAB model was attributed to the increased demand of the RV (Kheyfets et al., 2020), leading to less work being allocated to the systemic circulation. PAH induced in rats via MCT resulted in decreased LV and systemic pressures, and reduced LV $\frac{d\,p}{d\,t}$_max_ and $\frac{d\,p}{d\,t}$_min_, measured *in vivo* (Han et al., 2018). However, *ex vivo* testing of isolated LVs with Langendorff perfusion demonstrated the LV being capable of developing normal pressures at different afterloads (Han et al., 2018). Altered septal mechanics also contribute to LV dysfunction in the setting of PAH and RV dysfunction. In a clinical study, noninvasively determined regional myocardial function measured by echocardiographic myocardial strain was found to be reduced in the LV of pediatric PH patients and was largely related to decreases in septal strain (Burkett et al., 2015). These findings suggest that altered LV function in PAH is predominantly a result of the biomechanical interactions between the ventricles with alterations of septal mechanics, rather than intrinsic LV remodeling.

### Effects of PAH on Tissue-Level Biomechanics of RV Myocardium

#### Effects of PAH on Biaxial Mechanical Properties of the RVFW

**FIGURE 4:**
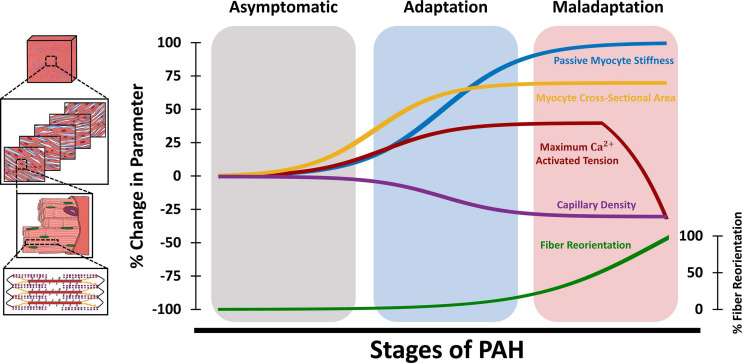
Schematic demonstration of changes in tissue, fiber, and myocyte-level RV biomechanical properties at different stages of PAH. The adaptation/maladaptation stages in this figure are defined based on the time-course of changes in organ-level hemodynamics (maintained/reduced cardiac output, ejection fraction, RV-PA coupling). The RV initially responds to elevated afterload in PAH via hypertrophy (increased myocyte cross-sectional area) and increased myocyte contractile forces (maximum Ca^2+^ activated tension), which results in increased organ-level contractility (E_es_). This is accompanied by myofiber stiffening (increased passive stiffness) and capillary rarefaction, leading to RV fibrosis. Passive myocyte mechanical properties, cross-sectional area and RV free wall capillary density remain relatively constant at later stages of PAH. The RV undergoes fiber reorientation with further progression of PAH, which is accompanied by impaired myocyte-level contractility and reduced ejection fraction and RV-PA coupling efficiency. RV: Right ventricle; PAH: Pulmonary arterial hypertension; PA: Pulmonary artery.

Biaxial mechanical testing remains the gold standard for evaluation of passive tissue-level RVFW biomechanical properties and has been employed in ex-vivo studies in small and large animal models (Valdez-Jasso et al., 2012; Hill et al., 2014; Javani et al., 2016; Nemavhola, 2017), as well as excised tissues from human donors (Sommer et al., 2015). While ex-vivo biaxial testing has been previously employed for characterization of active biomechanical properties of LV myocardium (Lin and Yin, 1998), biaxial studies on the RV have been mostly focused on the passive components of RVFW biomechanics. RVFW specimens from small and large animal models and human donors have demonstrated a nonlinear anisotropic biaxial response (Sacks and Chuong, 1993; Valdez-Jasso et al., 2012; Sommer et al., 2015). RVFW was shown to be stiffer and more anisotropic than the LV (Sacks and Chuong, 1993). The nonlinearity of the RVFW stress-strain response has been attributed to gradual collagen recruitment, in addition to nonlinear stiffening of myofibers (Avazmohammadi et al., 2017a), while RVFW fiber architecture accounts for tissue anisotropy. Diastolic filling was found to operate under stresses that result in RVFW strains below ≈5% (Jang et al., 2017), indicating the low-strain portion of the biaxial RVFW stress-strain curves (dominated by myofibers, before collagen recruitment) as the operational range of RV diastolic mechanics under normal physiological loading. The schematic in Figure 4 demonstrates the different tissue, fiber, and myocyte-level remodeling events during PAH progression. Also worth noting that, similar to Figure 2, the upper/lower bounds of changes in the biomechanical parameters presented in Figure 4 are based on previous reports in the literature (Supplementary Table 2), while the time-course of changes during the progression of PAH are illustrated based on a limited number of available longitudinal studies (Vonk Noordegraaf et al., 2017; Vélez-Rendón et al., 2018; Wang et al., 2018; Avazmohammadi et al., 2019a) and the authors’ speculation. This further reveals the need for multi-scale longitudinal studies to evaluate RV biomechanics during the progression of PAH. PAH results in increased biaxial RVFW stiffness, with a ≈2-fold increase in tissue anisotropy (Hill et al., 2014; Park et al., 2016). RV pressure overload in PAH has been shown to result in higher tissue-level stiffening in the longitudinal direction, due to RV fiber reorientation and alterations in the intrinsic mechanical properties of RVFW fibers (Hill et al., 2014; Park et al., 2016; Avazmohammadi et al., 2017a; Sharifi Kia et al., 2020). Increased RVFW stiffness in the longitudinal direction was correlated with increased organ-level diastolic elastance (E_ed_) in a PAB model of PAH (Jang et al., 2017). Passive RVFW stiffening was shown to initiate during the early stages of RV remodeling (Avazmohammadi et al., 2019a) and further increase with progression of PAH (Figure 4).

#### PAH-Induced RVFW Fibrosis

Quantitative histological analysis of collagen and myofiber area fractions is a commonly used technique to measure the tissue-level content of the RVFW. The RVFW extracellular matrix (ECM) mainly consists of fibrillar collagen networks (type I and III), basement membrane type IV collagen, elastin, proteoglycans, fibronectin, and laminin (Stroud et al., 2002). Healthy RVFW consists of 4-9% collagen (by area), depending on the tissue source and animal model (Javani et al., 2016; Akazawa et al., 2020; Sharifi Kia et al., 2020). However, despite relatively low area/volume fractions, the ECM plays a key role in passive RV biomechanics. Biaxial testing of decellularized RVFW specimens revealed that the ECM contributes to 23-26% of passive RV stiffness under ≈10% strains (Vélez-Rendón et al., 2019). Mechanical contribution of the ECM grows with increased collagen recruitment, contributing to 71-78% of biaxial RV stiffness at 30% strain (Avazmohammadi et al., 2017b). PAH has been shown to result in disrupted matrix turnover (Golob et al., 2016), altered matrix metalloproteinase (MMP) activity, and increased RVFW collagen content (Bogaard et al., 2009; Borgdorff et al., 2013; Andersen et al., 2014; Akazawa et al., 2020). RV fibrosis was noted in explanted tissues from PAH patients (Rain et al., 2013). A potential mechanism for RVFW fibrosis in PAH is capillary rarefaction (Borgdorff et al., 2013; Andersen et al., 2014; Akazawa et al., 2020), reduced RV perfusion and increased oxidative stress (Bogaard et al., 2009), leading to a mismatch between RVFW oxygen supply and demand. RV capillary density and myocardial perfusion was shown to decrease by ≈20-40% in early stages of PAH (Borgdorff et al., 2013; Wang et al., 2018; Akazawa et al., 2020) and remain relatively constant at later stages (Wang et al., 2018; Figure 4). Increased RVFW collagen content has been found to be correlated with organ-level diastolic dysfunction and reduced RV compliance (Cheng et al., 2018). Fibrosis may also lead to alterations in the dynamic viscoelastic properties of the RVFW tissue. In a large animal model of PAH, viscous damping constant of the RVFW (measure of viscoelasticity) was elevated in response to pressure overload (Stroud et al., 2002), while ex-vivo plasmin treatment of RVFW specimens lead to increased MMP-2 and MMP-9 activity, collagen degradation, and reduced viscous damping (Stroud et al., 2002).

### Effects of PAH on Fiber and Cellular-Level RV Architecture and Biomechanics

#### PAH-Induced Transmural RV Fiber Reorientation

The RVFW is formed by a transmural stack of myofibers and collagen with different orientations throughout the tissue thickness. Gradient-based image analysis techniques have been employed to analyze the transmural orientations of RV fibers from histological sections (Hill et al., 2014). In addition to quantitative histological analysis, other techniques such as diffusion tensor MRI (DT-MRI) and second-harmonic generation (SHG) microscopy can be utilized to assess the 3D helical architecture of RV myofibers (Nielsen et al., 2009; Sommer et al., 2015; Agger et al., 2016; Omann et al., 2019). However, long imaging times, availability, and cost-effectiveness, limit the applicability of DT-MRI for benchtop and clinical applications. While SHG microscopy provides high fidelity information on the microarchitecture of ventricular fibers, this technique has an imaging depth of <1.2 mm (Sommer et al., 2015). This limits SHG’s potential to study the full-thickness transmural fiber orientations of intact RV myocardium from large animal models and human donors, which are generally >2 mm thick (Javani et al., 2016; Nemavhola, 2017). RV fibers demonstrate counterclockwise rotations throughout the RVFW (Hill et al., 2014), with collagen fibers running nearly parallel to myofibers (Sharifi Kia et al., 2020). The transmural architecture of RV fibers leads to anisotropic properties at the tissue level and has the potential to modulate tissue and organ-level contractile function (Gomez et al., 2017; Avazmohammadi et al., 2019a). Transmural RV myofiber orientation range was found to decrease with body size, ranging from 110-120 degrees in small animal models (Nielsen et al., 2009; Avazmohammadi et al., 2017a) to 30-40 degrees in larger animals (Sacks and Chuong, 1993). PAH results in reorganization of the RVFW’s transmural fiber architecture, leading to a longitudinal shift in RV collagen and myofiber angles. This is accompanied by transmural reduction in fiber dispersions/splays, which results in increased tissue-level anisotropy (Hill et al., 2014; Park et al., 2016). In a mild, adaptive PAB model of PAH, no differences were observed in transmural RVFW fiber orientations (Nielsen et al., 2009). However, more severe PAB led to longitudinal reorientation of RV collagen and myofibers (Hill et al., 2014; Sharifi Kia et al., 2020), indicating fiber reorientation as an end-stage remodeling event in PAH (Figure 4). While PAH may result in intrinsic remodeling of the myocyte contractile apparatus (Fan et al., 1997; Wang et al., 2018), reorientation of RV myofibers has the potential to result in impaired organ-level contractility, RV-PA uncoupling, and reduced ejection fraction, independent of intrinsic changes in myocyte contractility (Avazmohammadi et al., 2019a). The underlying mechanisms of RV fiber reorientation, however, have remained largely unknown. While some studies have categorized fiber realignment as a compensatory event in PAH (Gomez et al., 2017), others have identified this as a maladaptive event, leading to RV failure (Avazmohammadi et al., 2019a). Parametric analysis of the effects of different remodeling events in chronic RV pressure overload demonstrated that changes in RV wall stress and stretch-induced deformations of the RVFW may lead to altered transmural RV fiber kinematics (Sharifi Kia et al., 2021). While concentric hypertrophy (increased wall thickness) demonstrated a potential protective role against longitudinal fiber realignment, it was shown that eccentric hypertrophy (RV dilation) may stimulate a longitudinal kinematic (deformation-induced) shift in RV fiber orientations (Sharifi Kia et al., 2021). Nevertheless, fiber reorientation due to altered RVFW deformations only represents one of the several candidates for fiber realignment in PAH. Other potential mechanisms (such as preferential hypertrophy: sarcomerogenesis of longitudinal fibers accompanied by degradation of circumferential fibers) require further exploration in future work to elucidate the biomechanical triggers leading to structural reorganization of the RVFW, and effects of these processes on organ-level RV function.

#### PAH-Induced Alterations in Active and Passive Myocyte-Level RV Mechanical Properties

Fiber and myocyte-level mechanical properties of RV myocardium are often characterized by direct measurements performed on isolated skinned (chemically permeabilized) trabeculae, to facilitate characterization of myocyte properties in the absence of mechanical contributions from the ECM. Mechanical characterizations are performed by attachment of ends of isolated trabeculae to force transducers and servo motors, while sarcomere length is monitored under a microscope. Passive and active (Ca^2+^ dependent) forces generated by RV trabeculae are then measured at different lengths, over a range of different Ca^2+^ concentrations. Passive mechanical properties of RV fibers can also be estimated from equibiaxial tissue-level responses of the RVFW (Fata et al., 2014; Hill et al., 2014). PAH has been shown to result in ≈30-50% increase in the maximum Ca^2+^ activated tension of RV myofilaments (Rain et al., 2013; Wang et al., 2018). Increases in myocyte Ca^2+^ activated tension manifest early in the progression of PAH (Wang et al., 2018) and remain at relatively constant levels before the onset of RV decompensation and maladaptive failure (Figure 4). Analyzing the biomechanical response of skinned trabeculae obtained from a mouse model of SuHx (Wang et al., 2018) showed increased Ca^2+^ sensitivity and peak tension with increased sarcomere length, at different timepoints over 56 days, demonstrating no impairment of the Frank-Starling mechanism. This has also been noted in large animal models of PAH due to hypobaric hypoxia (Walker et al., 2011), as well as isolated cardiomyocytes from PAH patients (Rain et al., 2013). Consistent reports from different studies on preserved cardiomyocyte Frank-Starling mechanism in PAH, even in the presence of organ-level dysfunction (Wang et al., 2018), is particularly interesting as this may indicate impaired organ-level function in PAH precedes myocyte-level contractile dysfunction. Further progression of PAH and end-stage RV remodeling, however, exhibits reduced myocyte peak Ca^2+^ activated tension (Figure 4). Following 250 days of PAB in rats, Fan et al. observed a 35% drop in myocyte peak tension, accompanied by reduced Ca^2+^ sensitivity and significant RV hypertrophy (Fan et al., 1997). Reduced calcium sensitivity is a major determinant of myocyte contractile dysfunction and has been attributed to PAH-induced alterations in phosphorylation patterns of several contractile proteins, including troponin I, troponin T, and myosin light chain 2 (Walker et al., 2011). Additionally, α and β myosin heavy chain (MHC) expressions are altered in RV pressure overload. A transition from α to β isoforms of MHCs has been noted in the RV in PAH, indicating maladaptive remodeling and depressed myocyte contractile dynamics (Andersen et al., 2014). A 34% reduction in RV myocyte α-MHC expression was observed in a MCT model of PAH, accompanied by decreased Ca^2+^ activated ATPase activity (Vescovo et al., 1989). The transition to slower MHC isoforms (α→β) manifested in reduced contractile shortening amplitude and decreased speed of shortening in MCT animals (Vescovo et al., 1989). It should be noted that there are differences in myosin between species, such as differences in the relative expressions of α and β (healthy human hearts are predominantly β MHC while murine hearts are α predominant), as well as functional differences (for example murine myosin has greater contractile velocity than larger species with less difference in force generation between α and β (Malmqvist et al., 2004)). Myocyte mitochondrial function is another important key player in RV contractile biomechanics and development of RV failure in PAH. PAH has been shown to result in decreased RV mitochondrial volume density, while showing an increase in total number of mitochondria, indicating a mismatch between the rates of RV growth and mitochondrial biogenesis (Liu et al., 2017b). Transmission electron microscopy of RV specimens obtained from rats, in a PAB model of PAH, showed an increase in mitochondrial mass with decreased cross-sectional areas (accumulation of small-sized mitochondria), resulting in elevated oxygen consumption and decreased mechanical efficiency (Cheng et al., 2018).

α and β-adrenergic receptors (α-AR and β-AR) on cardiomyocytes play a crucial role in regulating myocardial morphology and contractility. β-AR stimulation is primarily responsible for increased cardiac performance under pressure overload, while α-ARs contribute to myocardial hypertrophy (Chen et al., 1999). RVFW biopsies from a large animal MCT model indicated that α and β-AR receptor density was significantly increased in response to PAH, manifesting in organ-level adaptive remodeling via increased systolic function and Fulton index (Chen et al., 1999). Cardiomyocyte hypertrophy and increased cross-sectional area were noted in both experimental models of PAH (Fan et al., 1997; Borgdorff et al., 2013; Andersen et al., 2014; Wang et al., 2018) and explanted RV samples from PAH patients (Rain et al., 2013). Myocyte cross-sectional area was shown to increase by 40-100% (Borgdorff et al., 2013; Rain et al., 2013; Wang et al., 2018; Akazawa et al., 2020) during early-stage RV adaptations, and remain relatively constant with further progression of PAH (Figure 4). Myocyte width, however, was shown to be increased only at late stages of PAH, as a morphological marker of RV dysfunction (Fan et al., 1997; Wang et al., 2018).

In addition to alterations in active myocyte contractile properties, pressure overload in PAH results in a progressive increase in passive stiffness of myofibers by ≈2 folds (Rain et al., 2013; Wang et al., 2018; Sharifi Kia et al., 2020), most of which has been shown to occur during the early stages of RV remodeling (Wang et al., 2018; Figure 4). This was associated with RV sarcomere stiffening with similar titin isoform composition to controls, but significantly reduced phosphorylation (Rain et al., 2013). Incubation of skinned myocytes with actomyosin inhibitor 2,3-butanedione monoxime did not demonstrate any effects on passive myocyte biomechanical properties, indicating that increased passive stiffness in PAH is potentially independent of actin-myosin interactions (Rain et al., 2013). PAH also results in a denser and thicker network of collagen fibers in the RVFW (Stroud et al., 2002; Vélez-Rendón et al., 2019) with reduced crimp, leading to earlier engagement of collagen fibers under passive loading (Avazmohammadi et al., 2017a; Sharifi Kia et al., 2020).

### Effects of Potential Therapeutics on RV Biomechanics in PAH

Despite recent developments of several therapeutics for management of PH, so far, lung transplantation remains the only curative treatment. While multiple clinical and preclinical studies have investigated the effects of potential therapeutics on RV function in PAH, only a few have analyzed the effects of these interventions on the biomechanics of the RV.

The renin-angiotensin-aldosterone system (RAAS) plays a major role in regulating RV remodeling in response to pressure overload (Menendez, 2016). In response to decreased systemic pressures, a cascade of reactions leads to upregulation of angiotensin II which then, through AT1 receptor signaling, results in vasoconstriction, sodium and water retention, and increased blood pressures. Previous work has evaluated the effects of angiotensin II receptor blockers on RV remodeling in PAH, by targeting the RAAS system and inhibiting the effects of AT1 receptor stimulation, the increase of vascular tone, and vasoconstriction. While angiotensin II receptor blockers have shown improvements in the setting of LV pressure overload, they did not demonstrate any effects on either compensated or decompensated RV failure, measured by RV pressures, cardiac output, dilation, contractility, and survival (Borgdorff et al., 2013; Andersen et al., 2014; Clements et al., 2019). Another major regulator of RV remodeling in PAH is the natriuretic peptides (NP) system. RV pressure overload has been shown to result in increased expression of atrial and brain natriuretic peptides (ANP and BNP, respectively); stress/stretch-dependent markers of cardiac hypertrophy and RV dysfunction (Andersen et al., 2014). NPs have the potential to result in vasodilation and reduced blood pressures (Menendez, 2016). However, inactive NP products are degraded by several peptidases, including neprilysin (Menendez, 2016), which hampers the protective effects of the NP system in PAH. While isolated angiotensin receptor blocking has not shown much improvements in PAH, simultaneous targeting of the RAAS and the NP system via combined angiotensin receptor blocking and neprilysin inhibition with Sacubitril/Valsartan (Sac/Val) has been shown to result in reduced RV pressures, RV hypertrophy, and pulmonary vascular wall thickness, in addition to maintained stroke volume and improved organ-level contractile and relaxation function (Clements et al., 2019; Sharifi Kia et al., 2020). Sac/Val prevents vasoconstriction and the degradation of inactive NP products, which leads to improved RV function. Sac/Val treatment prevented RV-PA uncoupling and maladaptive RV failure in SuHx and PAB models of PAH (Clements et al., 2019; Sharifi Kia et al., 2020) and was shown to result in reduced BNP levels in the RVFW (Clements et al., 2019), potentially via decreasing RV afterload/wall stress. At the tissue level, Sac/Val treatment was shown to result in reduced biaxial stiffness in the circumferential and longitudinal directions (Sharifi Kia et al., 2020). Sac/Val also attenuated passive myofiber stiffening and prevented transmural reorientation of RV collagen and myofibers (Sharifi Kia et al., 2020). Whether these preclinical results will bear out clinically remains to be seen.

In addition to conventional pathways for management of heart failure, a number of studies have analyzed the potential of other therapeutic targets and interventions in PAH. Integrin-linked kinase (ILK) is an enzyme upregulated in PA vascular smooth muscle cells in PAH, resulting in PA remodeling and proliferation. ILK inhibition was shown to result in reduced PA and RV hypertrophy, decreased RV systolic and end-diastolic pressures, and improved contractility in male, but not female rats in a SuHx model (Shen et al., 2020). This was attributed to estrogen-mediated effects in females, as co-culture of human PA vascular smooth muscle cells with estrogen and ILK inhibitors, hindered the anti-proliferative effects of ILK inhibition (Shen et al., 2020). Nevertheless, ILK inhibition could be a potential therapeutic target for management of PAH, at least in males. Additionally, placement of mesenchymal stem cell (MSC)-seeded bioscaffolds on rat RVs in a SuHx model resulted in improved stroke volume, cardiac output, and diastolic function, despite showing no effects on RV systolic pressures, $\frac{d\,p}{d\,t}$_max and min_, and RV-PA coupling efficiency (Schmuck et al., 2019). Reduced RV fibrosis and myocyte hypertrophy, in addition to increased coronary perfusion, were among the potential mechanisms of action for the observed effects.

### *In silico* Modeling of RV Biomechanics in PAH

*In silico* modeling of RV biomechanics in PAH has gained significant traction in recent years. Computational models can provide valuable insights into the underlying mechanisms of RV remodeling in PAH and can help better understanding the time-course of different remodeling events. *In silico* modelling facilitates parametric analysis of the relative contribution of isolated remodeling events to RV dysfunction; something that would be otherwise time-consuming or impractical to measure experimentally. The multi-scale *in silico* models used to study RV function range from lumped parameter hemodynamic models of the pulmonary vasculature (Grant and Paradowski, 1987) to organ and tissue-level models incorporating 3D animal/patient-specific biventricular geometries (Finsberg et al., 2019), structurally detailed fiber-level models (Gomez et al., 2017), and cell/molecular-level models coupling mitochondrial mechanoenergetics to cross-bridge cycling and cardiomyocyte contractile dynamics (Pewowaruk et al., 2018). For a detailed review on multi-scale modeling of RV-PA mechanics in PAH, please refer to the recent work by Vélez-Rendón and Valdez-Jasso (2019).

3D biventricular models were used to study the effects of PAH on RVFW fiber stress (Xi et al., 2016; Scardulla et al., 2018), demonstrating a 2-7 fold increase in fiber stress in PAH patients. In another study, strain analysis using biventricular models generated from PAH patients indicated a significant reduction in all 3 components of RV contractile strains (circumferential, longitudinal, and radial) compared to controls (Finsberg et al., 2019; Zou et al., 2020). This was accompanied by decreased myofiber contractile shortening strain (Finsberg et al., 2019), which showed a strong negative correlation with the ratio of RV to LV end-diastolic volumes (RVEDV/LVEDV). Patients with RVEDV/LVEDV ≥ 1.5 demonstrated 25% lower shortening strains, suggesting an association between RV dilation and contractile dysfunction (Finsberg et al., 2019). 3D strains were shown to be better markers of RV dysfunction compared to conventional markers such as 2D strains or ejection fraction. Gomez et al. used biventricular models informed with myocardial fiber architectures (measured via DT-MRI) to study the effects of fiber reorientation on RV function in a PAB model of PAH (Gomez et al., 2017). With all other parameters kept constant, longitudinal fiber remodeling was found to result in decreased end-systolic volumes, leading to increased ejection fraction. Moreover, longitudinal fiber reorientation reduced the myofiber contractility required to maintain a target cardiac output. Based on this evidence, the authors classified fiber reorientation as an adaptive remodeling event in PAH, resulting in a boost in RV ejection fraction (Gomez et al., 2017). In another structurally informed biventricular model of RV remodeling in a MCT model of PAH, a significant increase in Ca^2+^ activated myocyte tension was noted, in addition to tissue stiffening, and longitudinal fiber reorientation (Avazmohammadi et al., 2019b). RV hypertrophy normalized the circumferential components of RV wall stress in PAH, while longitudinal stress remained significantly higher than controls. This suggested a link between elevated longitudinal wall stress and realignment of RVFW fibers. Further investigation using a growth and remodeling framework indicated that fiber reorientation led to disruption of the optimal RVFW fiber architecture (Avazmohammadi et al., 2019a). Despite increased myocyte-level contractility, the non-optimal architecture resulting from fiber realignment, led to impaired organ-level function and reduced ejection fraction (more contractility at the myocyte-level, but reduced contractility at the organ-level). This led to categorization of longitudinal RV fiber reorientation as a maladaptive event in PAH (Avazmohammadi et al., 2019a), in contrast to a previous report suggesting an adaptive role for fiber remodeling (Gomez et al., 2017). Biventricular FE modeling of RV function in pediatric PAH also showed a slight decrease in RV ejection fraction with longitudinal fiber remodeling, accompanied by decreased LV twisting motions and elevated LV myocardial stress (Kheyfets et al., 2019).

Coupling biventricular simulations with models of the PA enables parametric assessment of the effects of PA remodeling on RV biomechanics and RV-PA coupling (Tewari et al., 2013; Shavik et al., 2020). *In silico* analysis of PA mechanics in a SuHx model predicted a 110% increase in PA elastance accompanied by a 130% elevation in PA resistance and RVFW stiffening (Tewari et al., 2013). In a recent computational study by Shavik et al. (2020) increasing proximal PA collagen content in the setting of PAH resulted in increased PA pulse pressures and decreased PA compliance, leading to elevated RV afterload and systolic pressures. However, increasing distal PA resistance showed a more significant effect on RV and PA pressures compared to proximal PA fibrosis, suggesting that microcirculation remodeling may have a more crucial role in increased RV afterload in PAH than remodeling of the proximal PA (Shavik et al., 2020). This is consistent with long standing clinical observations that PAH involves small vessel vascular remodeling (Shepherd et al., 1957; Edwards and Edwards, 1977; Humbert et al., 2019). Recently Pewowaruk et al. (2018) used a multi-scale modeling approach to analyze RV function in PH. Mitochondrial mechanoenergetics and actin-myosin cross-bridge cycling were coupled to tissue and organ-level mechanics to simulate RV, LV, and PA hemodynamics. Computational modeling revealed the capability of the RV to maintain cardiac output in a scenario of isolated pressure overload with no remodeling (Pewowaruk et al., 2018). However, including the effects of decreased myofiber contractility and altered metabolic energetics, led to reduced cardiac output and RV-PA uncoupling (Pewowaruk et al., 2018), indicating a role for peak myocyte contractile forces and metabolite concentrations in regulating RV adaptation vs. maladaptation.

## Discussion

Clinically, outcomes in PAH are related to RV structure and function. The concept of detailed risk assessment in PAH, particularly focused on the RV, is promoting earlier and more aggressive treatment of patients (Galiè et al., 2019). The study of the RV is thus highly clinically relevant. Tissue biomechanics links cellular and subcellular mechanisms to organ-level observations, and can provide insights that may be clinically translated to diagnostics and therapeutics. For example, increases in passive RV tissue stiffness has been correlated with organ-level diastolic dysfunction (Jang et al., 2017), and diastolic dysfunction has been linked to poor clinical outcomes (Vanderpool et al., 2015). Myofiber reorientation has been linked to increased tissue anisotropy and RV-PA uncoupling, which in turn has been linked to clinical outcomes. Tissue biomechanics can be a platform to study the potential treatments targeting the RV, and may elucidate novel therapeutic targets. The ability to adapt complex measures of tissue biomechanics and function to noninvasive imaging can allow for widespread clinical translation. Insights gained experimentally can be used for *in silico* modeling, which can be a faster and more cost-effective approach to guide future preclinical and clinical research.

Despite promising advances in understanding RV remodeling in PAH, there remains a continued need for further studies on the biomechanical effects of these remodeling events, to facilitate the design and personalization of potential therapeutics aiming to induce reverse remodeling. Potential directions for future work include:

(1) Long-term longitudinal biomechanical studies looking at the time-course of different multi-scale remodeling events in PAH.
(2) Biomechanical evaluation of the underlying mechanisms of fiber reorientation in PAH, elucidating the effects of fiber realignment on organ-level function.

(3) Biomechanical analysis of the adaptiveness of different RV remodeling events in relation to function, and characterization of the transition points from adaptation to maladaptation.
(4) Identification of a biomechanical metric for characterization of the “point of no return” in PAH progression, beyond which the RV progresses into failure
(5) Biomechanical analysis of RV function in other phenotypes of PH, including PH-LHD.

## Author Contributions

DS drafted the manuscript and generated the figures. KK drafted the manuscript. MS drafted the manuscript and provided clinical perspective on the review topic. All authors reviewed the final manuscript.

## Conflict of Interest

DK is employed by Align Technology, Inc. MS was on the consultation/scientific advisory board for Bial, Acceleron, Altavant; and executed hemodynamic core lab work for Aadi. The remaining author declares that the research was conducted in the absence of any commercial or financial relationships that could be construed as a potential conflict of interest.

## Abbreviations

α-AR and β-AR: α and β Adrenergic Receptors
$\frac{\text{dp}}{\text{dt}}\,\text{max}$ and $\frac{\text{dp}}{\text{dt}}\,\text{min}$, Load-Dependent Measures of RV Contractility and Relaxation: Respectively
cMRI: Cardiac Magnetic Resonance Imaging
DT-MRI: Diffusion Tensor Magnetic Resonance Imaging
E_a_: Pulmonary Artery Elastance (Approximate Measure of RV Afterload)
ECM: Extracellular Matrix
EDPVR: End-Diastolic Pressure-Volume Relationship
E_ed_: Instantaneous End-Diastolic Elastance
E_es_: End-Systolic Elastance (Load-independent measure of RV Contractility)
ESPVR: End-Systolic Pressure-Volume Relationship
ILK: Integrin-Linked Kinase
LV: Left Ventricle
MCT: Monocrotaline
MHC: Myosin Heavy Chain
MMP: Matrix Metalloproteinases
mPAP: Mean Pulmonary Artery Pressures
NP: Natriuretic Peptides
PA: Pulmonary Artery
PAB: Pulmonary Artery Banding
PAH: Pulmonary Arterial Hypertension
PH: Pulmonary Hypertension
PH-LHD: Pulmonary Hypertension due to Left Heart Disease
PVR: Pulmonary Vascular Resistance
RAAS: Renin-Angiotensin-Aldosterone System
RV: Right Ventricle
RVFW: Right Ventricular Free Wall
Sac/Val: Sacubitril/Valsartan
SHG Microscopy: Second-Harmonic Generation Microscopy
SuHx: Sugen-Hypoxia.
