# Supplementary material for: Current Understanding of the Right Ventricle Structure and Function in Pulmonary Arterial Hypertension
Source: Front Physiol. 2021 May 28;12:641310. doi: 10.3389/fphys.2021.641310 (PMC8194310; doi:10.3389/fphys.2021.641310)
Supplement: Supplementary file 1 [file Data_Sheet_1.PDF]

Supplementary Material

1 Supplementary Tables

Supplementary Table 1: PAH-induced alterations in organ-level RV functional parameters, used to generate Figure 2 in the main body of the manuscript

Parameter	PAH	Control	References	Maximum %Change in Fig. 2
**mPAP**	47 mmHg	16 mmHg	(Rain et al., 2013)	↑200%
	58 mmHg	-	(Vanderpool et al., 2020)	
	42 mmHg	-	(Tello et al., 2020)	
**E_{es}**	1.65 mmHg/mL	0.48 mmHg/mL	(Gaynor et al., 2005)	↑200%
	1.1 mmHg/mL	0.4 mmHg/mL	(Rain et al., 2013)	
	10.2 mmHg/RVU	3.6 mmHg/RVU	(Sharifi Kia et al., 2020)	
**RV Wall Thickness**	1.29 mm	0.66 mm	(Jang et al., 2017)	↑100%
	1.12 mm	0.61 mm	(Vélez-Rendón et al., 2018)	
	1.34 mm	0.66 mm	(Sharifi Kia et al., 2020)	
**RV End-Diastolic Volume (Index)**	35 µL	24 µL	(Wang et al., 2018)	↑50%
	77.5 µL	40.1 µL	(Avazmohammadi et al., 2019)	
	109 mL/m^2	73 mL/m^2	(Zou et al., 2020)	

**Ejection Fraction**	36%	57%	(Rain et al., 2013)	↓35%
	29%	45%	(Kaiser et al., 2020)	
	45%	70%	(Cheng et al., 2018)	
**RV-PA Coupling**	0.41	0.71	(Sharifi Kia et al., 2020)	↓50%
	0.4	0.75	(Wang et al., 2018)	
**Cardiac Output**	150 mL/min	220 mL/min	(Lahm et al., 2016)	↓50%
	70 mL/min	120 mL/min	(Cheng et al., 2018)	
	25 mL/min	125 mL/min	(Akazawa et al., 2020)	

Supplementary Table 2: PAH-induced alterations in tissue and myocyte-level RV biomechanics, used to generate Figure 4 in the main body of the manuscript

Parameter	PAH	Control	References	Maximum %Change in Fig. 4
**Passive Myocyte Stiffness**	≈10 kPa	≈5 kPa	(Rain et al., 2013)	↑%100
	168 kPa	81 kPa	(Sharifi Kia et al., 2020)	
	10 kPa	5.5 kPa	(Wang et al., 2018)	
**Myocyte Cross-Sectional Area**	480 μm^2	240 μm^2	(Rain et al., 2013)	↑%70
	26 μm^2	18 μm^2	(Borgdorff et al., 2013)	
	1000 μm^2	500 μm^2	(Akazawa et al., 2020)	
	350 μm^2	250 μm^2	(Wang et al., 2018)	
**Maximum Ca^{2+} Activated Tension**	24 kPa	16 kPa	(Rain et al., 2013)	↑%40 → ↓%35
	44 kPa	35 kPa	(Wang et al., 2018)	
	27 kPa (End-Stage)	42 kPa	(Fan et al., 1997)	
**Capillary Density**	22.5 Capillaries per 0.01 mm^2	31 Capillaries per 0.01 mm^2	(Borgdorff et al., 2013)	↓%30
	4.75%	6%	(Akazawa et al., 2020)	
	5%	9%	(Wang et al., 2018)	
